# Kritische Erfolgsfaktoren für ideales Arbeiten im Home-Office – eine empirische Analyse aus Arbeitgeber- und Arbeitnehmersicht

**DOI:** 10.1365/s40702-022-00880-x

**Published:** 2022-05-31

**Authors:** Maik Jeske, Andreas Schmidt, Andreas Gadatsch

**Affiliations:** Hochschule Bonn-Rhein-Sieg, Sankt Augustin, Deutschland

**Keywords:** Home-Office, Anforderungen, Erfolgsfaktoren, Digitalisierung, Effekte, Home-Office, Requirements, Challenges, Digitalization, Effects

## Abstract

Vor dem Hintergrund der Covid-19-Pandemie hat sich das Home-Office in Deutschland seit dem Jahr 2020 weit verbreitet und wird seitdem bei vielen Arbeitgebern als neue Arbeitsmethode genutzt. Der Einsatz von Home-Office kann verschiedene positive als auch negative Effekte auf die Beschäftigten und den Arbeitgeber sowie die Gesellschaft allgemein haben. Damit von möglichst vielen positiven Effekten profitiert werden kann, ist ein gutes Home-Office Konzept erforderlich. Welche Anforderungen an ein solches Konzept bestehen und welche Voraussetzungen grundlegend mit der Nutzung von Home-Office verbunden sind, wird in dem Beitrag aufgezeigt. Dabei werden von technischen bis hin zu sozialen Aspekten Anforderungen verschiedener Arten berücksichtigt, welche durch eine durch den Autor durchgeführte Studie gebildet worden sind. Im Fokus dieses Beitrages sollen die kritischen Erfolgsfaktoren für das ideale Arbeiten im Home-Office stehen, also die Anforderungen, welche ausschlaggebend für die erfolgreiche Umsetzung eines Home-Office Konzeptes sind und Einfluss auf die wahrgenommenen Effekte des Home-Office haben. Die im Beitrag aufgeführte Studie des Autors wurde im Rahmen der Abschlussarbeit von Herrn Jeske durchgeführt, auf welcher der Beitrag basiert.

## Abstract (en)

Against the background of the Covid-19 pandemic, the home office has become widespread in Germany since 2020 and has since been used as a new working method by many employers. The use of home offices can have various positive and negative effects on employees and employers as well as society in general. In order to benefit from as many positive effects as possible, a good home office concept is required. The article shows which requirements exist for such a concept and which prerequisites are fundamentally associated with the use of home offices. From technical to social aspects, requirements of various types are considered, which have been formed by a study carried out by the author. The focus of this article should be the critical success factors for ideal work in the home office, i.e. the requirements that are decisive for the successful implementation of a home office concept and have an influence on the perceived effects of the home office. The author’s study listed in the article was carried out as part of Mr. Jeske’s thesis, on which the article is based.

## Einleitung

Im Jahr 2020 hat sich unsere Lebensweise und somit unsere Arbeitsweise durch die anhaltende Coronapandemie grundlegend geändert. Das Home-Office gewinnt stark an Bedeutung und ist zum momentanen Zeitpunkt nicht aus der Wirtschaft wegzudenken. Durch staatliche Schließungen und Maßnahmen waren viele Unternehmen während der Covid-19 Pandemie dazu gezwungen, ihre Arbeit ins Home-Office zu verlegen (vgl. IfSG § 28b Abs. 7 2021). Eine solche Umstellung erfordert je nach Branche und Fortschritt des Unternehmens viel Arbeit und Investitionen. Durch gegebene Faktoren wie die Infrastruktur, den Digitalisierungsstand der Branche sowie die Bereitschaft der Mitarbeiter, sich an die neue Arbeitsweise anzupassen, ist es interessant zu erfahren, inwiefern Arbeitgeber^1^ in der Lage sind, umgehend ideale Arbeitsbedingungen im Home-Office zu schaffen.

Vor dem Hintergrund des New-Work Konzeptes und dessen Werte kann das Home-Office eine neue Arbeitsmethode für die zukünftige Arbeitswelt darstellen. Der New-Work Ansatz stammt vom österreichisch-amerikanischen Philosophen Fritjof Bergmann und ist bereits über 40 Jahre alt (vgl. Jobst-Jürgens 2020, S. 2). Dieses ursprüngliche Konzept sieht ein innovatives Arbeitsumfeld vor, in dem die persönlichen Werte und Selbstverwirklichungswünsche des Einzelnen berücksichtigt werden. Dazu müssen sich alte Strukturen wie ein striktes Hierarchiesystem, feste Arbeitszeiten und -orte und die Führung durch Ansagen auflösen und durch neue entsprechende Methoden ersetzt werden. Werte wie Freiheit, Zufriedenheit und Selbsterfüllung sollen in den Fokus gerückt werden, sodass sich nicht nur die Art der Arbeit, sondern die gesamte Art zu leben ändert, denn der Mensch werde durch vorgeschriebene, nicht erfüllende Arbeit zermürbt. Durch die Verwendung von neuer Technik sollen unannehmliche Lohnarbeiten verringert werden und den Menschen soll mehr Zeit verschafft werden, um einem höheren Ziel nachzugehen (vgl. Jobst-Jürgens 2020, S. 2). Während das Konzept zuvor als eine utopische gesellschaftliche Veränderung angesehen wurde, wird New Work heutzutage vielmehr als Sammelbegriff für verschiedene neue Arbeitsformen und -methoden als zentraler Bestandteil von sinnstiftender und freier Arbeit genutzt (vgl. Jobst-Jürgens, S. 4). So wird der Begriff häufig mit weiteren Schlagworten wie Arbeiten 4.0, Digitalisierung und auch dem Home-Office in Verbindung gesetzt (vgl. Berend und Brohm-Brady 2020, S. 12).

## Grundlagen und Effekte von Home-Office

### Gesetzliche Regelungen

Um die gesetzlichen Regelungen zum Home-Office darlegen zu können, ist zunächst eine Abgrenzung ähnlicher Begriffe erforderlich. Es sollte grundlegend in Telearbeit und mobiles Arbeiten unterschieden werden (vgl. Anabah 2021). Beide Begriffe werden zwar im Zusammenhang mit Home-Office genannt, unterscheiden sich jedoch in ihren Einzelheiten. Während bei der Telearbeit seitens des Arbeitgebers ein fester Arbeitsplatz im privaten Raum der Beschäftigten geschaffen wird, werden diese bei dem Konzept des mobilen Arbeitens lediglich in dem Maße ausgestattet, dass sie ortsunabhängig also auch unterwegs arbeiten können (vgl. CMS Legal Services EEIG (Hrsg.) 2021). Bei Einsatz von Telearbeit muss die Arbeitsstättenverordnung, die Arbeitszeitverordnung sowie das Datenschutzgesetz berücksichtigt werden (vgl. Haufe (Hrsg.) 2021). Bei mobilem Arbeiten, ohne festen Arbeitsplatz, muss die Arbeitsstättenverordnung hingegen nicht beachtet werden, da der Arbeitgeber hier weniger verantwortlich für die Ausstattung am Arbeitsplatz ist und nur bedingt Einfluss darauf nehmen kann (vgl. Wolf und Kring 2021). Das Arbeitsschutzgesetz und das Arbeitszeitgesetz müssen bei mobilem Arbeiten ebenfalls eingehalten werden (vgl. CMS Legal Services EEIG (Hrsg.) 2021).

Grundsätzlich existiert in Deutschland bislang kein gesetzlicher Anspruch auf die Arbeit im Home-Office (vgl. Anabah 2021). Im Verlauf der Covid-19 Pandemie kam es jedoch wiederholt zur Einführung einer zeitweisen Homeoffice-Pflicht zwecks Eindämmung des Pandemiegeschehens, welche zuletzt als Erweiterung des § 28b des Infektionsschutzgesetzes am 24. November 2021 (vgl. Infektionsschutzgesetz (IfSG) vom 20. Juli 2000 (2000)) wieder in Kraft getreten ist und voraussichtlich bis zum 19. März 2022 gilt. Der Verpflichtung ist jedoch nur nachzukommen, sofern von Arbeitgeber- als auch von Arbeitnehmerseite keine betriebsbedingten Gründe dagegenstehen (vgl. Bundesamt für Arbeit und Soziales (BMAS) (Hrsg.) 2022).

### Aktuelle Arbeitgeberperspektiven zum Home-Office Einsatz

Um einen Einsatz von Home-Office in Erwägung zu ziehen, sollte zunächst beachtet werden, ob ein Arbeitgeber grundlegend dazu geeignet ist. Denn: Nicht alle Branchen und Tätigkeiten lassen eine Arbeit im Home-Office zu. Im Rahmen des kununu Corona Employer Transparency Ticker wurden über 31.000 Beschäftigte verschiedener Branchen aus Deutschland, Österreich und der Schweiz zum Umgang ihres Arbeitgebers mit dem Thema Home-Office befragt. Dabei wurde nachgefragt, ob Arbeit im Home-Office grundsätzlich möglich ist und wie es vergleichsweise in derselben Branche gehandhabt wird (vgl. kununu GmbH (Hrsg.) 2020).

Es ist zu beobachten, dass es viele Branchen gibt, welche schlicht keine Möglichkeit zur Arbeit im Home-Office besitzen. Dabei handelt es sich unter anderem um die Pflege- und Gesundheitsbranche, die Textilindustrie, Transportunternehmen, Gastronomie- und Tourismusunternehmen sowie Handwerksbetriebe. Es wird deutlich, dass Home-Office in diesen praxisintensiven Branchen aufgrund der Tätigkeitsbeschreibungen der zugehörigen Arbeitsplätze nicht umsetzbar ist. Zwar gibt es in der Studie Zahlen, die zeigen, dass ein Teil der Beschäftigten dieser Branchen im Home-Office arbeiten könnte, jedoch ist bei diesen Fällen anzunehmen, dass es sich um vereinzelte Büroarbeitsplätze, beispielsweise in der Unternehmensverwaltung, handelt. Gegenteilig zu den genannten Branchen ist zu erkennen, dass bürointensive Branchen viel mehr Potenzial zur Einrichtung von Home-Office-Arbeitsplätzen haben. So haben über 80 % der Befragten in der IT- oder Internetbranche während der Coronapandemie von Zuhause aus gearbeitet. In der IT-Branche hätten bis zu 93 % der befragten Beschäftigten von Zuhause aus arbeiten können. Auch in der Versicherungsbranche gaben rund 80 % der Befragten an, dass sie das Home-Office-Angebot ihres Arbeitgebers nutzen während bei bis zu 88 % ein Angebot zur Arbeit im Home-Office vorlag. Auch bei den Steuerberatern und Steuerberaterinnen könnten 87 % der Befragten im Home-Office arbeiten. Hier ist die Abweichung zur tatsächlichen Nutzung sehr hoch. Nur 59 % der Befragten haben die Möglichkeit wahrgenommen. Welche Gründe dies hat, wurde in der Befragung nicht untersucht. Ableiten kann man jedoch trotzdem, dass die reine Möglichkeit zur Arbeit im Home-Office nicht unmittelbar zur Wahrnehmung dieser führt (vgl. kununu GmbH (Hrsg.) 2020).

Mögliche Gründe für die teils großen Unterschiede der Home-Office Nutzung zwischen den Branchen könnten demnach auch in den verschiedenen Tätigkeiten begründet sein. So sollten die Tätigkeiten der Beschäftigten in verschiedene Gruppen unterteilt und somit in mehrere Arten von Arbeit unterschieden werden. Die erste Gruppe umfasst analytische Tätigkeiten, im Sinne von Büroarbeit mit hohem Anteil an Schreibarbeiten, Denkprozessen und Bedienung von IT-Systemen. Die zweite Gruppe beinhaltet alle interaktiven Tätigkeiten, bei denen die Zusammenarbeit mit anderen Menschen im Fokus steht. Hierzu zählen zum Beispiel Pflegeberufe oder Lehrtätigkeiten. Die letzte Gruppe bezieht sich auf die manuellen Tätigkeiten. Diese umfassen alle Aufgaben, die mit körperlicher Anstrengung verbunden sind, typischerweise Handwerksberufe (vgl. Leibniz-Institut für Bildungsverläufe e.V (Hrsg.) 2021, S. 3). Während Branchen mit hohem Anteil an analytischen Tätigkeiten ein hohes Home-Office-Potenzial besitzen, sinkt dieses mit der Zunahme von interaktiven und vor allem manuellen Tätigkeiten. Dies ist dadurch zu begründen, dass beispielsweise Personen mit überwiegend interaktiven Tätigkeiten oft kaum auf den direkten Kontakt zu ihren Kunden, Patienten und ähnlichen verzichten können. Ebenso verhält es sich mit Personen, welche manuelle Tätigkeiten ausführen, da dort nicht selten Maschinen und andere stationäre Einrichtungen erforderlich sind (vgl. Grunau et al. 2019, S. 5).

Neben den genannten Einflüssen hat auch die Digitalisierungsstand eines Unternehmens Auswirkungen auf die Home-Office Nutzung bei Unternehmen, Behörden und anderen Organisationen. Der Zusammenhang dieser beiden Punkte wurde im Jahr 2020 durch eine Umfrage des Bundesamtes für Sicherheit in der Informationstechnik genauer untersucht. Die Ergebnisse der Umfrage zeigen, dass Beschäftigte, welche die Möglichkeit zur Arbeit im Home-Office besitzen, den Digitalisierungsstand ihres Arbeitgebers tendenziell besser bewertet haben (vgl. Bundesamt für Sicherheit in der Informationstechnik (Hrsg.) 2021, S. 19). Daraus lässt sich schließen, dass der Digitalisierungsstand eines Unternehmens durchaus als Indikator für die Home-Office Nutzung verwendet werden kann.

Des Weiteren können Unternehmen, Behörden und andere Organisationen in drei Gruppen hinsichtlich ihres Digitalisierungsstandes in Abhängigkeit ihrer Nutzung von digitalen Arbeitsweisen eingeteilt werden. Digitale Vorreiter führen neue Methoden schnell ein und nutzen diese trotz der Gefahr, etwas falsch zu machen. Durchschnittlich digitale Unternehmen warten zunächst die Erfahrungen der digitalen Vorreiter ab und führen entsprechend im Nachgang erfolgreiche Arbeitsmethoden bei sich selbst ein. Die digitalen Nachzügler verzichten weitestgehend auf die Einführung neuer digitaler Arbeitsweisen und halten an bestehenden Prozessen fest (vgl. DAK Gesundheit (Hrsg.) 2020, S. 6). Während digitale Vorreiter und die durchschnittlich digitalen Organisationen zum Einsatz von Home-Office tendieren, lehnen digitale Nachzügler den Einsatz von eben diesem eher ab (vgl. DAK Gesundheit (Hrsg.) 2020, S. 7).

### Erfahrungen aus der Nutzung von Home-Office

Durch die Nutzung von Home-Office konnten Beschäftigte, Arbeitgeber und ebenfalls weitere Teile der Gesellschaft neue positive als auch negative Effekte wahrnehmen, welche durch herkömmliche Arbeit bislang nicht oder nicht in diesem Maße aufgetreten sind. Dazu haben sich bereits verschiedene Studien mit beschäftigt, deren wichtigste Erkenntnisse folgend knapp zusammengefasst werden.

Der in den Umfragen am häufigsten genannte positive Effekt der Arbeit im Home-Office ist die Erleichterung der Vereinbarkeit zwischen Familie und Beruf (vgl. Hans-Böckler-Stiftung (Hrsg.) 2020). Sofern die Mitarbeiter ihre Arbeitszeiten flexibel einteilen dürfen, können private wie berufliche Termine besser geplant und Kinder vermehrt betreut werden, wobei zu beachten ist, dass Frauen bis zu drei Stunden mehr mit Kinderbetreuung verbringen, wenn Sie im Home-Office arbeiten. Männer hingegen arbeiten stattdessen tendenziell mehr. (vgl. Lott 2019, S. 5ff.). In Bezug auf die Arbeitszufriedenheit kann beobachtet werden, dass Personen, die flexible Arbeitsmodelle nutzen, insgesamt nur geringfügig zufriedener sind als Beschäftigte, die fest vor Ort arbeiten. Es kann allerdings zu einem umgekehrten Effekt kommen. Wenn Beschäftigte, welche den Wunsch besitzen, von Zuhause aus zu arbeiten und bei denen dies auch aufgrund der Tätigkeit möglich wäre, keine Möglichkeit zur Arbeit im Home-Office erhalten, so sinkt deren Arbeitszufriedenheit im Vergleich signifikant ab (vgl. Brenke 2016, S. 103).

Für Arbeitgeber kann der Einsatz von Home-Office grundsätzlich als Chance angesehen werden. Mit der Einführung von Home-Office oder anderen Formen mobiler Arbeit ist es erforderlich, entsprechende IT-Infrastrukturen auszubauen und neue digitale Arbeitsmethoden einzuführen. Diese ermöglichen über das Home-Office hinaus neue Wege zur besseren Arbeit (vgl. Initiative D21 (Hrsg.) 2021). Im Hinblick auf den Digitalisierungsstand eines Unternehmens, einer Behörde oder Ähnlichem kann die Einführung von Home-Office durchaus zu einem regelrechten Digitalisierungsschub führen (vgl. DAK Gesundheit (Hrsg.) 2020, S. 7). Zugleich könnte hier andererseits eine Gefahr für sehr schlecht digitalisierte Arbeitgeber, die digitalen Nachzügler, bestehen, hinter den Konkurrenten zurückzubleiben, da diese, wie in Abschnitt 2.2 beschrieben, tendenziell weniger neue Arbeitsmethoden von sich aus einführen und nutzen (vgl. DAK Gesundheit (Hrsg.) 2020, S. 7). Der erforderliche Ausbau der IT-Infrastruktur und das Einsetzen neuer digitaler Arbeitsmethoden erhöht jedoch zeitgleich den Bedarf am Ausbau der IT-Sicherheit eines Unternehmens (vgl. Bundesamt für Sicherheit in der Informationstechnik (Hrsg.) 2021, S. 3). Grund dafür sind die erhöhte Anzahl an externen Verbindungen und der Einsatz unternehmensexterner Anwendungen, welche zusätzlichen Schutz benötigen. Oft wird private IT-Ausstattung zur Arbeit verwendet, welche ein erhöhtes Risiko, die Datensicherheit zu verletzen, besitzt. Zusätzlicher Schutz kann durch die Verwendung von VPN-Verbindungen, Mehrfach-Faktor-Authentifizierungen und gesonderten Schutzmaßnahmen für mobile Endgeräte gewährleistet werden (vgl. Bundesamt für Sicherheit in der Informationstechnik (Hrsg.) 2021, S. 8f.). Ebenso können Gefahren durch Cyberangriffe durch die Sensibilisierung der Mitarbeiter zum Thema Datensicherheit vermindert werden (vgl. Bundesamt für Sicherheit in der Informationstechnik (Hrsg.) 2021, S. 11). Besonders für kleine Unternehmen mit weniger als 50 Beschäftigten und Unternehmen mit einem schlechten Digitalisierungsstand können Cyberangriffe existenzbedrohende Ausmaße annehmen, jedoch besitzen diese meist keine speziellen Schutzmaßnahmen für das Home-Office (vgl. Bundesamt für Sicherheit in der Informationstechnik (Hrsg.) 2021, S. 14).

Neben weiteren positiven Effekten wie einer geringfügigen Steigerung der Produktivität der Beschäftigten im Home-Office (vgl. DAK Gesundheit (Hrsg.) 2020, S. 11) haben Unternehmen außerdem die Möglichkeit ihre Attraktivität als potenzielle Arbeitgeber zu erhöhen (vgl. Grunau et al. 2019, S. 4). So wurden im Jahr 2019 Studierende deutscher Universitäten im Rahmen einer experimentellen Studie dazu aufgefordert, aufgrund von Stellenausschreibungen die Attraktivität verschiedener Arbeitgeber zu bewerten. Arbeitgeber, welche in ihren Stellenausschreibungen flexible Arbeitsformen wie Home-Office angeboten haben, haben durchschnittlich bessere Bewertungen durch die Versuchspersonen erhalten. Das Angebot von Home-Office kann demnach einen großen Vorteil für Arbeitgeber auf dem hart umkämpften Arbeitsmarkt darstellen (vgl. Schmoll und Süß 2019, S. 50ff.). Des Weiteren könnte der Einsatz von Home-Office in Kombination mit weiteren neuen Arbeitskonzepten wie Desk-Sharing und Co-Working-Spaces eine Einsparung an Büroflächen bewirken. Arbeitgeber hätten dadurch die Möglichkeit künftig Kosten in Form von Miete, Wartung und Ausstattung zu verringern (vgl. Klaffke (Hrsg.) 2016, S. 3f.).

Eingesparte Büroflächen können anschließend alternativ genutzt werden. Dabei müssen diese nicht zwingend durch den entsprechenden Arbeitgeber genutzt werden, sondern kann anderen Interessensgruppen zugutekommen. So können eingesparte Büroflächen zu Wohnraum umfunktioniert werden. Besonders in Deutschland besteht eine große Nachfrage nach bezahlbarem Wohnraum, insgesamt fehlt es an ausreichend Wohnungen (vgl. pestel Institut (Hrsg.) 2019, S. 3). Es wäre möglich bis 2025 rund 235.000 zusätzliche Wohnungen durch die Umfunktionierung von Büroflächen zu schaffen (vgl. Zeit Online (Hrsg.) 2021). Auch eine Verringerung der Umweltbelastung durch die Bewältigung von Arbeitswegen kann durch den vermehrten Einsatz von Home-Office bewirkt werden. Eine Studie von Greenpeace hat dazu zwei Szenarien entwickelt, welche darstellen wieviel CO2 sich durch die Arbeit im Home-Office in Abhängigkeit zur Anzahl der Beschäftigten einsparen ließe. Es wird unterschieden zwischen einem konservativen Szenario, bei dem 25 % der Beschäftigten die Möglichkeit haben im Home-Office zu arbeiten, und einem erweiterten Szenario, bei welchem der Anteil der potenziellen Home-Office-Nutzer 40 % der arbeitenden Bevölkerung umfasst. Zudem wird angenommen, dass sich diese Anteile der Beschäftigten langfristig nicht jeden Tag im Home-Office befinden. Daher wurde berechnet, wieviel CO2 sich mit einem Home-Office-Anteil von einem oder zwei Tagen pro Arbeitswoche einsparen ließe (vgl. Büttner und Breitkreuz 2020, S. II). Im konservativen Szenario könnten rund 5 % der jährlichen Emissionen des Pendelverkehrs eingespart werden, während der Anteil beim erweiterten Szenario auf 11 % steigen würde (vgl. Büttner und Breitkreuz 2020, S. 14).

Neben den genannten positiven Effekten hat die zunehmende Arbeit im Home-Office jedoch auch negative Folgen für bestimmte Bereiche der Gesellschaft. So kommt es vor allem in Branchen wie der Reinigungsbranche, der Gastronomie sowie der Textilindustrie zu starken wirtschaftlichen Rückgängen, da diese infolge der Zunahme der Home-Office Nutzung weniger Aufträge und Kunden erhalten. Dies ist damit verbunden, dass ein beträchtlicher Teil der Kundschaft der benannten Branchen durch die Arbeit im Home-Office nicht weiter auf die Dienstleistung oder das Produkt der Unternehmen angewiesen ist (vgl. Atradius Kreditversicherung (Hrsg.) 2021).

## Empirische Studie

### Ziele und Gegenstand

Ziel der empirischen Studie war die Beantwortung der Frage, welche Anforderungen an das ideale Arbeiten im Home-Office bestehen und welche Effekte die Arbeit im Home-Office auf Beschäftigte, Arbeitgeber und die Gesellschaft allgemein haben kann. Die zur Beantwortung der Frage verwendete Definition von „idealem Arbeiten“ lautet wie folgt: Die Anforderungen an das ideale Arbeiten im Home-Office, umfassen zum einen die Anforderungen, welche bestehen, um ein möglichst büroähnliches Arbeiten zu ermöglichen, jedoch zeitgleich die positiven Effekte des Home-Office bewahren, während negative Auswirkungen weitestgehend durch bestimmte Anforderungen beziehungsweise Maßnahmen verringert werden sollen.

### Methodik

Um die zuvor genannte Frage zu beantworten, wurden zunächst leitfadengestützte, halbstandardisierte Interviews (vgl. Hussy et al. 2013, S. 224) mit den Beschäftigten einer mittelgroßen Steuerkanzlei geführt. Ziel der Interviews war es, die Mitarbeiter zu ihren Erfahrungen im Home-Office zu befragen, aktuelle Probleme im Home-Office zu erkennen und erste Thesen über die Effekte und den damit verbundenen Anforderungen an die Arbeit im Home-Office zu formulieren.

Bei der Erstellung des Interviewleitfadens wurde darauf geachtet, die Fragen möglichst offen zu stellen, um den Befragten Spielraum für ihre Antworten zu geben und unterschiedlich starke Wahrnehmungen bestimmter Effekte und die persönlichen Situationen der Befragten besser aufnehmen zu können. Die Kanzlei wurde für die Interviews ausgewählt, da hier sichergestellt werden konnte, dass alle Befragten bereits ausgiebige Erfahrungen im Home-Office sammeln konnten. Außerdem verfügt das Unternehmen über eine bereits gut ausgebaute technische Infrastruktur, sodass eventuell Probleme über die grundlegenden technischen Voraussetzungen hinaus erkannt werden konnten. Allen geeigneten im Unternehmen tätigen Personen wurde angeboten, an einem Interview teilzunehmen. Dabei wurden alle Personen im Vorhinein über den Zweck der Interviews sowie die anschließende Weiterverwendung der gewonnenen Daten aufgeklärt. Nach diesem Vorgehen wurden insgesamt fünf Personen befragt, welche unterschiedlichen Altersgruppen angehören, verschiedene Positionen im Unternehmen einnehmen und sich in verschiedenen privaten Situationen befinden. Die Interviews wurden aufgrund der aktuellen Hygieneschutzmaßnahmen, bedingt durch die Covid-19 Pandemie, online mithilfe der Kommunikationsplattform Microsoft Teams durchgeführt. Alle Interviews fanden im Zeitraum vom 30.03.2021 bis zum 06.04.2021 statt und haben im Schnitt 30 min pro befragter Person gedauert. Während der Interviews befanden sich die Befragten jeweils allein in abgeschlossenen Räumen und auch im Onlinemeeting waren ausschließlich die befragte Person und der Interviewer anwesend. So konnte weitestgehend ausgeschlossen werden, dass die Personen durch sozialen Druck von außen bei ihren Antworten beeinflusst worden wären. Die Interviews wurden nach Unterschreiben einer Einverständniserklärung mithilfe der Aufnahmesoftware OBS Studio aufgezeichnet, wobei lediglich die Tonspuren aufgenommen worden sind. Dies hatte den Grund, dass die meisten Interviewteilnehmer sich mit der Aufnahme Ihres Bildes unwohl gefühlt haben und nicht damit einverstanden waren. Während der Durchführung der Interviews gab es keine sonstigen Störfaktoren.

Die aufgezeichneten Interviews wurden anschließend mithilfe einer qualitativen Inhaltsanalyse nach Mayring ausgewertet (vgl. Krüger et al. 2014, S. 135ff.). Dazu wurden zunächst schriftliche Transkripte der einzelnen Interviews nach den Regeln von Dresing und Pehl in der einfachen Variante angefertigt. Sprachfehler, Dialekte sowie Handlungen durch Gestik und Mimik wurden nicht detailliert erfasst (vgl. Dresing und Pehl 2012, S. 21ff.). Ebenso wurden bei den Interview Transkripten die Einleitung, welche viele personenbezogene Daten enthielt, entfernt. Bei der qualitativen Inhaltsanalyse nach Mayring wurde nach einem induktiven Vorgehen gearbeitet, es wurden also während der Analyse der Interviews Themen-Kategorien gebildet (vgl. Krüger et al. 2014, S. 139). Im Anschluss wurden in diese Kategorien diverse Aussagen der Befragten in Form einer Kategoriensystemtabelle zugeordnet und paraphrasiert. Daraufhin folgte die Auswertung der aufbereiteten Aussagen in den verschiedenen Kategorien und verfolgte als Ziel, die Bildung von Thesen und Hypothesen über die Effekte und Anforderungen von Home-Office.

Um die gebildeten Thesen und Hypothesen zu überprüfen, wurde eine quantitative Studie in Form eines Onlinefragebogens durchgeführt. Dazu wurden zu den Thesen passende Fragen mit einem ordinalen Skalenniveau in Form von 7er-Likert-Skalen (vgl. Statista (Hrsg.) 2021) entwickelt. Vor der Versendung der Umfrage wurde diese mithilfe eines Pre-Tests auf ihre Funktion und untersucht und mehrfach überarbeitet, um die Fragen verständlicher zu machen.

Die Umfrage wurde am 18.04.2021 online gestellt und über die Social-Media Plattformen Facebook und Instagram durch mehrere private Benutzerkonten geteilt und verbreitet. Um möglichst viele Personen verschiedener Berufsgruppen und Branchen zu befragen, wurde die Umfrage zudem in diversen Unternehmen verteilt und weiterverbreitet. Auch eine Steuerkanzlei hat zur Verbreitung der Umfrage beigetragen, indem der Link zur Umfrage durch die Geschäftsführer an diverse Mandate der Steuerberatungskanzlei versendet worden ist. Es wurde auch darauf geachtet, Unternehmen verschiedener Größen zu berücksichtigen und auch Bundesämter und damit Beamte in die Umfrage miteinzubeziehen. Die Umfrage wurde ebenfalls über einen E‑Mailverteiler der Hochschule Bonn-Rhein-Sieg verbreitet und somit an eine Vielzahl von Studierenden weitergeleitet, welche gegebenenfalls einer Nebentätigkeit im Home-Office nachgehen oder zuvor in einem Beschäftigungsverhältnis mit der Möglichkeit zur Arbeit im Home-Office standen. Dies wurde überprüft, indem die Umfrageteilnehmenden gefragt worden sind, ob sie aktuell einer beruflichen Tätigkeit im Home-Office nachgehen oder ob sie dies innerhalb der letzten drei Jahre getan haben. In diesem Sinne werden die Teilnehmenden im Folgenden als „Beschäftigte“ zusammengefasst. Teilnehmende, welche keiner Tätigkeit im Home-Office nachgingen, flossen nicht in die Gruppe der Beschäftigten ein. Insgesamt machten Angestellte, mit 28,3 %, und Studierende mit 65,4 % den größten Anteil der Teilnehmenden aus. Die restlichen Teilnehmenden verteilten sich auf Selbstständige, Auszubildende und Sonstige. Rentner und Arbeitssuchende waren nahezu nicht in der Studie vertreten. Die Online-Umfrage stand vom 18.04.2021 bis zum 21.05.2021 zur Verfügung. Während dem Umfragezeitraum nahmen insgesamt 779 Personen an der Umfrage teil. Jedoch wurden alle Antwortbögen von der Auswertung ausgeschlossen, wenn diese entweder nicht vollständig ausgefüllt waren, eindeutig falsch ausgefüllt oder die Teilnehmenden nicht mit der Datenschutzerklärung einverstanden waren. Durch eine Beantwortungsrate von 75,73 % und den zuvor genannten Bedingungen kam eine Stichprobe der Größe *n* = 590 zustande. Da die Onlineumfrage überwiegend in Unternehmen und weiteren Arbeitgebern in Nordrhein-Westfalen verteilt worden ist, sind die Umfrageergebnisse ebenfalls nur auf den genannten geographischen Raum zu beziehen. Auch ist die hohe Anzahl an Studierenden unter den Teilnehmenden zu berücksichtigen. Ebenso ist das niedrige Durchschnittsalter von 24 Jahren zu beachten. Die Aufteilung der Teilnehmenden wird folgend grob dargestellt. 365 Teilnehmende sind der Altersgruppe von 18 bis 25 Jahren zuzuordnen, 141 Teilnehmende der Altersgruppe von 26 bis 39 Jahren und 84 Teilnehmende der Altersgruppe von 40 bis 66 Jahren. Bei der Auswertung wurde das Alter der Teilnehmenden jedoch weitestgehend nicht berücksichtigt, da sich auf Grundlage der geführten Interviews nur wenig altersabhängige Annahmen ergeben haben.

Die in der Umfrage erhobenen Daten wurden anschließend mithilfe von SPSS auf Korrelationen mit der Korrelationsanalyse nach Kendall-Tau‑b (vgl. Cohen 1988, S. 78ff.) untersucht und die aufgestellten Thesen somit überprüft. Auf Grundlage dessen wurden Anforderungen an die Arbeit im Home-Office abgeleitet.

## Ergebnisse der Studie

### Anforderungen an den Arbeitgeber

Grundsätzlich müssen viele der folgend genannten Anforderungen an die persönlichen Situationen der Beschäftigten eines Arbeitgebers angepasst werden. Denn, so ergab die Studie, Beschäftigte, die beispielsweise außerhalb der Arbeit keine oder wenige soziale Kontakte haben, fühlen sich eher einsam als Beschäftigte mit vielen privaten Kontakten. Auch die berufliche Position ist stets zu berücksichtigen. Auszubildende haben der Studie zufolge häufiger Probleme mit der Arbeit im Home-Office als andere Beschäftigte. So waren ebenfalls Beschäftigte mit Kindern im Home-Office stärker in Hinblick auf ihre Produktivität beeinträchtigt als Beschäftigte ohne Kinder. Eine tiefergehende Berücksichtigung verschiedener Einflüsse in Abhängigkeit des Familienstandes darüber hinaus wurde in der Studie nicht vorgenommen, da während den geführten Interviews lediglich der Aspekt von Kindern im Home-Office seitens der Befragten genannt worden ist.

Den Ergebnissen der Umfrage zufolge ist es seitens des Arbeitgebers notwendig Maßnahmen zu ergreifen, um der sozialen Isolation der Beschäftigten z. B. in Form von Freizeitangeboten vorzubeugen. Das diese ein großes Problem darstellt, wird dadurch deutlich, dass über 50 % der Befragten der Aussage zustimmten, sich im Home-Office einsam zu fühlen. Des Weiteren sagten über 83 % der Befragten, dass sie im Home-Office deutlich weniger Kontakt zu ihren Kollegen haben. Die durch das Home-Office geschaffene Distanz zwischen den Beschäftigten unter sich und auch zu den Führungskräften muss zwingend vereinfacht werden, im idealen Fall in einer Form, in der man ohne Einschränkungen miteinander arbeiten kann. Zu diesem Zweck ist die Einführung entsprechender Kommunikationssoftware in einem Unternehmen einer der ersten zu bewältigenden Schritte. Um auf bestimmte Probleme individuell eingehen zu können, sollte stets ein offener Umgang mit den Beschäftigten geführt und aktiv nachgefragt werden, wie sich das soziale Wohlbefinden des Einzelnen steigern lässt. Ein konsequenter Einbezug der Beschäftigten im Home-Office ist nötig, um deren Zugehörigkeitsgefühl zum Arbeitgeber aufrecht zu erhalten. Dies kann in Form von wöchentlichen Meetings bis hin zur Integration bei Entscheidungen stattfinden. Ein weiterer Vorteil von regelmäßigen Treffen, ist die damit verbundene Informationsweitergabe. Rund 73,5 % der Befragten stimmten dem zu und sind der Ansicht, dass regelmäßige Treffen den Zusammenhalt fördern würden. Der Arbeitgeber bleibt so transparent gegenüber seinen Beschäftigten, obwohl durch das Home-Office eine Distanz geschaffen wird.

Die Arbeit im Home-Office besitzt auch insbesondere für Führungskräfte weitere Anforderungen. Eine Führungskraft, welche Beschäftigte im Home-Office führen muss oder selbst aus dem Home-Office führt, sollte stets gut erreichbar sein oder klar kommunizieren, wann ein Austausch mit ihr möglich ist. Bei größeren Führungsebenen sollten demnach klare Zuständigkeiten in Form von festen Ansprechpartnern festgelegt werden.

**Abb. 1:**
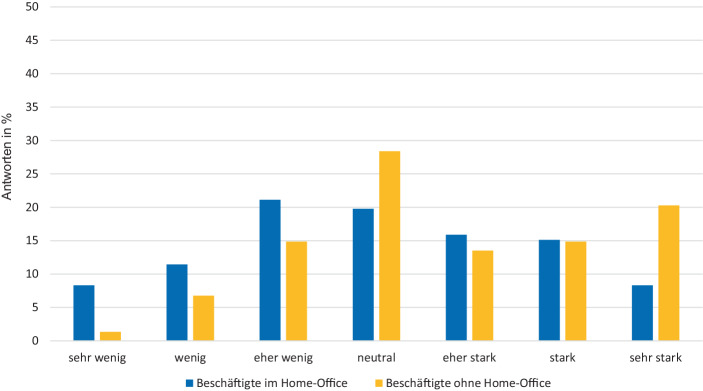
Ergebnisse der Frage nach Einhaltung der Arbeitszeiten. „Wie sehr halten Sie sich an Ihre üblichen Arbeitszeiten in Hinblick auf Beginn und Ende?“ (Quelle: Eigene Darstellung. Daten entnommen aus eigener empirischer Erhebung)

In der Studie war zu beobachten, dass es im Home-Office vermehrt zu Mehrarbeit kommt. Wie in Abb. 1 dargestellt fällt es Beschäftigten im Home-Office tendenziell schwerer ihre üblichen Arbeitszeiten einzuhalten, wodurch es zu Überstunden kommen kann. Bezogen auf Letzteres antwortete knapp ein Drittel der Befragten, dass sie im Home-Office mehr oder deutlich mehr arbeiten würden als vor Ort. Dies erfordert eine genauere Absprache zwischen Arbeitgebern und Beschäftigten in Hinblick auf die Arbeitszeitregelungen, um Beschäftigte davor zu schützen zu viel zu arbeiten. So sollten die genauen Arbeitszeiten zum Schutze der Beschäftigten auch für den Arbeitgeber nachvollziehbar sein, was jedoch damit einhergehen könnte, dass der Vorteil der Flexibilität für die Beschäftigten zu einem gewissen Grad verloren ginge.

**Abb. 2:**
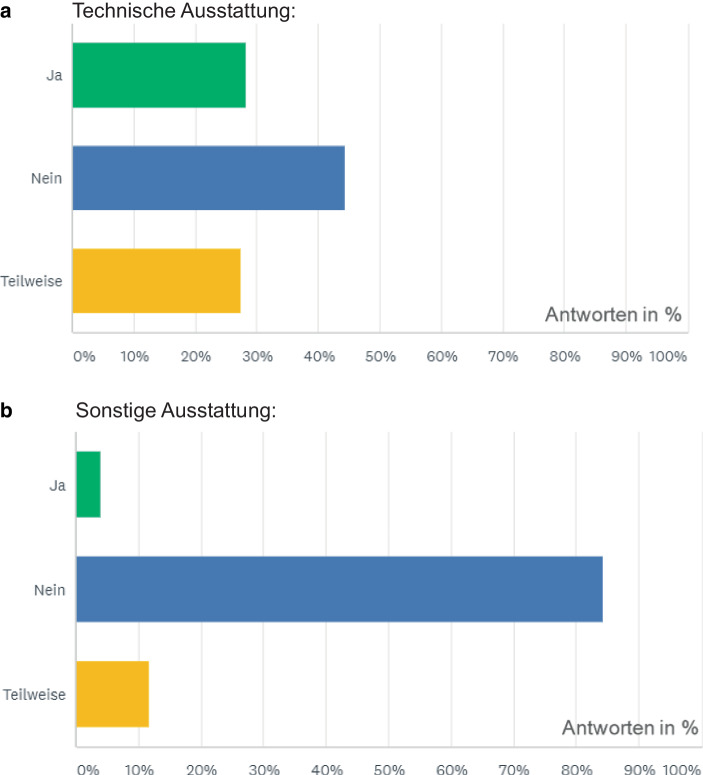
Ergebnisse der Frage nach Bereitstellung von Ausstattung. „Wird die Ausstattung für das Home-Office von Ihrem Arbeitgeber gestellt?“ (Quelle: Eigene Darstellung. Daten entnommen aus eigener empirischer Erhebung)

Hinsichtlich des Arbeitsplatzes im Home-Office ist eine gewisse Grundausstattung bestehend aus allen zur Arbeit nötigen Geräten, Materialien, Software-Anwendungen sowie Möbeln erforderlich. Oft fehlt es bislang an mindestens einem der genannten Punkten. So bemängeln rund 15 % aller Teilnehmenden, dass sie keinen geeigneten PC besitzen. 20 % fehlen kleine Peripheriegeräte wie eine Computermaus oder Tastaturen. Allein bei Druckern und Scannern gibt es bei der technischen Ausstattung einen großen Bedarf. 48 % der Beschäftigten vermissen einen Drucker oder einen Scanner an ihrem Home-Office-Arbeitsplatz. Ein weiterer, häufig genannter Punkt ist das Fehlen von Möbelstücken, zum Beispiel Schreibtische oder Bürostühle. Über 55 % aller Befragten fehlt mindestens ein Möbelstück im Home-Office. Auch sonstige Arbeitsmaterialen wie zum Beispiel Papier oder Büroklammern fehlen in über 25 % der Fälle. Wenn jedoch essenzielle Ausstattung fehlt, kommt es zu Hindernissen in den Arbeitsabläufen. Auch andere Studien haben bereits herausgefunden, dass eine Grundausstattung im Home-Office einen ergonomischen Arbeitsplatz, ausreichende Hardwareausstattung inklusive Telefon, Internetverbindung und Software umfassen sollte (vgl. Bruhn 2020, S. 1f.). Besonders die zur Verfügungstellung von Möbelstücken wird bei vielen Arbeitgebern bislang nicht berücksichtigt, dabei könnte besonders dadurch die Gesundheit der Beschäftigten besser geschützt oder sogar gefördert werden, zum Beispiel durch ausfahrbare Stehtische. Selbstverständlich sollten jederzeit alle Vorschriften der Arbeitsstättenverordnung eingehalten werden. Wie häufig die technischen und sonstigen Arbeitsausstattungen von den Arbeitgebern gestellt werden, ist folgend in Abb. 2 dargestellt.

Einen sehr bedeutenden Einfluss auf die Möglichkeiten zur Arbeit im Home-Office und damit eine der wichtigsten Anforderungen ist der Digitalisierungsstand des Arbeitgebers. Dieser sollte sich auf einem Niveau befinden, auf dem alle notwendigen Voraussetzungen erfüllt sind, um neue Technologien und digitale Arbeitsprozesse ohne Probleme umsetzen zu können. Besonders digitale Nachzügler sind dazu häufig nicht in der Lage und müssen für den idealen Einsatz von Home-Office dringend Verbesserungen vornehmen. Auch alle weiteren Arbeitgeber müssen einen kontinuierlichen Verbesserungsprozess der eigenen Technologien anstreben. Denn ohne ausreichende IT-Infrastruktur drohen, wie unter Punkt 2.3 beschrieben negative Effekte in Hinblick auf den Datenschutz und die Produktivität der Beschäftigten. Ergänzend dazu ist zu erwähnen, dass bei über 80 % der Befragten die Arbeit digitalisierbar ist. Nur unter zwei Prozent der interviewten Personen gaben an, dass ihre Arbeit nicht digitalisierbar sei. Auch das Abhalten entsprechender Schulungen im Umgang mit neuer Technik ist eine an den Arbeitgeber gestellte Anforderung. Durch die Studie wurde festgestellt, dass Beschäftigte mit Erfahrungen im Home-Office und weiteren Kenntnissen, wie einer weitreichenden Technikaffinität, besser im Home-Office arbeiten können als Beschäftigte, die sich im Vergleich dazu wenig mit den genannten Themen auskennen. Besonders für die Behebung und Vermeidung von technischen Problemen sollte der Einsatz einer eigenen IT-Abteilung diskutiert und auch dabei eine klare Zuständigkeit vermittelt werden.

### Anforderungen an die Beschäftigten

Die Anforderungen an die Beschäftigten bestehen vor allem in Anforderungen an deren Arbeitsweise und Einstellung gegenüber ihrer Arbeit. Für die Arbeit im Home-Office sollten Beschäftigte vor allem selbstdiszipliniert und eigenmotiviert arbeiten können, da sie im Home-Office wenig kontrolliert werden können. Arbeitsabläufe müssen selbstständig geplant werden während Ablenkungen und Störfaktoren weitestgehend ausgeschlossen werden sollten. Zur Überprüfung der Produktivität, Effizienz und Selbstdisziplin wurden mehrere Fragen gestellt, welche jeweils anhand von verschiedenen Merkmalen, z. B. der Selbsteinschätzung der eigenen Konzentrationsfähigkeit im Home-Office, die Arbeitsweise in Hinblick auf die Produktivität erfragt haben. Die Selbstdisziplin eines Beschäftigten und dessen Effizienz weisen einen mittelstarken positiven Zusammenhang (*r* = 0,321; *p* < 0,001) nach Kendall-Tau‑b auf. Dahingehend kann die These bestätigt werden, dass eine geringere Selbstdisziplin zu einer verringerten Effizienz bei der Arbeit im Home-Office führt. Die Fähigkeit zur Selbstreflexion ist ebenfalls bedeutend, um feststellen zu können, wie gut man selbst tatsächlich im Home-Office arbeiten kann. Des Weiteren müssen Beschäftigte im Home-Office offen für Neuerungen sein. Dies umfasst die Nutzung neuer Technologien bis hin zur Weiterbildung des eigenen Wissenstandes sowie gesammelte Erfahrungen, um optimal auf die Arbeit im Home-Office vorbereitet zu sein. Durch die statistische Untersuchung der Studienergebnisse konnte ein Zusammenhang zwischen bisherigen Erfahrungen im Home-Office und der Produktivität eines Beschäftigten sowie dem technischen Kenntnisstand eines Beschäftigten und der Produktivität nachgewiesen werden.

Die Beschäftigten müssen sich zudem Gedanken zur Einrichtung ihres Arbeitsplatzes machen. Da der Arbeitgeber kaum Einfluss auf die privaten Räumlichkeiten der Beschäftigten hat, liegt es in deren Verantwortung einen möglichst geeigneten Raum zum Arbeiten zu organisieren. Die Studie zeigt auf, dass dies bei rund 60 % der Teilnehmenden ein Problem darstellt und sie keinen separaten Raum zum Arbeiten haben. Besonders junge Menschen im Alter von 18 bis 24 Jahren scheinen häufig kein separates Arbeitszimmer zu besitzen. Die Verfügbarkeit von diesem steigt mit dem Alter stetig an, so sind es überwiegend Personen im Alter ab 40 Jahren, die ein eigenes Arbeitszimmer besitzen. Ein Grund hierfür könnte sein, dass junge Menschen in Deutschland durchschnittlich erst mit fast 24 Jahren aus dem Elternhaus ausziehen und somit erst dann eine eigene Wohnung besitzen (vgl. Eurostat (Hrsg.) 2021). Da ein großer Anteil der Teilnehmenden in der Altersgruppe von 18 bis 25 Jahren liegt, war der hohe Anteil an Personen ohne separates Arbeitszimmer in Hinblick auf die oben genannte Statistik zu erwarten. An dieser Stelle wäre eine nähere Betrachtung der Größe der Wohnflächen der Beschäftigten interessant gewesen. Dieser Aspekt wurde in der Studie jedoch nicht weiter untersucht. Des Weiteren müssen Aspekte wie die Möglichkeit zur sicheren Aufbewahrung von sensiblen Daten sowie die Nutzung von ausschließlich betrieblichen Geräten beachtet werden. Letzteres könnte den Beschäftigten die Trennung von Arbeit und Freizeit erleichtern, indem Arbeitsmittel in dafür vorgesehenen Orten verwahrt werden. Denn rund 67 % der Befragten gaben an, durch Arbeitsmittel im privaten Raum Arbeit und Freizeit schlechter trennen zu können.

Ergänzend zu den genannten Anforderungen hat die Studie ebenfalls die Wahrnehmung vieler in Kapitel 2.3 genannten positiven Effekte auf die Beschäftigten bestätigt. So nannten über 80 % der Befragten den Vorteil der zeitlichen Flexibilität. Auch die Zeitersparnis, z. B. durch das Wegfallen anfallender Anfahrten wurde mit knapp 70 % häufig genannt. 36 % der Befragten gaben sogar an, Familie und Beruf durch die Arbeit im Home-Office besser miteinander vereinbaren zu können. Ebenso nahmen 56 % der Befragten eine Kostenersparnis durch die Arbeit im Home-Office wahr. Welche Kosten hierbei explizit eingespart wurden, könnte über die Studie hinaus genauer untersucht werden.

### Kritische Erfolgsfaktoren

Wie im vorangegangenen Abschnitt 4.1 bereits angedeutet, können die Anforderungen an ideales Arbeiten im Home-Office grob in grundlegende Anforderungen, welche die Arbeit im Home-Office erst ermöglichen, und optionale Anforderungen, welche die Qualität der Arbeit im Home-Office positiv beeinflussen, unterteilt werden. Die meisten der grundlegenden Anforderungen können hierbei auch als die auschlaggebenden, kritischen Erfolgsfaktoren gesehen werden, da deren Einhaltung zwingend für ein gutes Arbeiten im Home-Office nötig ist.

Zu den kritischen Erfolgsfaktoren für das ideale Arbeiten im Home-Office zählt allen voran die Arbeitsausstattung. Diese sollte alle Mittel umfassen, die ein Beschäftigter benötigt, um seine Arbeit möglichst effizient und angenehm zu bewältigen. So sollten sämtliche Arbeitsmittel vom Arbeitgeber gestellt werden und von Arbeitsmaterialien, über die technische Ausstattung bis hin zu Möbeln wie beispielsweise ergonomischen Stühlen alles umfassen. Durch die Studie ließ sich ableiten, dass sich die Produktivität der Beschäftigten durch eine höhere Zufriedenheit mit der eigenen Arbeitsausstattung positiv beeinflussen lässt. Auch die beim Arbeitgeber eingesetzte Software ist ein wichtiger Bestandteil eines funktionierenden Home-Office Konzeptes. So sollte den Beschäftigten ein Mittel zur Kommunikation in Form einer Softwareanwendung zur Verfügung stehen, da diese die Kommunikation im Home-Office vereinfachen kann. 94 % der in der Studie Befragten gaben an, eine Kommunikationssoftware zu nutzen, wobei rund 69 % der insgesamt Befragten angaben nie oder nur sehr selten Kommunikationsprobleme zu haben. Anknüpfend daran ist ebenfalls der allgemeine Digitalisierungsstand eines Arbeitgebers ein kritischer Erfolgsfaktor für das ideale Arbeiten im Home-Office. Neben einer Verringerung des Risikos von Cyberangriffen mit steigender IT-Sicherheit, wie in Abschnitt 2.3 beschrieben, bildet die technische und digitale Infrastruktur eines Arbeitgebers die Grundlage für die Einrichtung von Home-Office Arbeitsplätzen, welche zunehmend in Form von Digital Workplaces realisiert werden. Ein Digital Workplace (übersetzt: digitaler Arbeitsplatz) ist eine digitale Softwareumgebung, in der alle zur Arbeit nötigen Funktionen integriert sind. Dazu zählen E‑Mail-Systeme, Cloudanwendungen, Dokumentenmanagementsysteme und viele Weitere (vgl. Ashri 2020, S. 76). In diesem Zusammenhang ist zu schlussfolgern, dass Beschäftigte entsprechend ausreichend schnelle Internetanschlüsse benötigen, um überhaupt im Home-Office arbeiten zu können. Wie im vorangegangenen Kapitel beschrieben ist zudem ein separates Arbeitszimmer von Vorteil, um möglichst produktiv im Home-Office arbeiten zu können.

Der wohl bedeutendste Erfolgsfaktor ist der Einfluss der sozialen Aspekte, allen voran die Gefahr der sozialen Isolation durch die Arbeit im Home-Office. Wie in Abschnitt 4.1 zuvor beschrieben, fühlen sich besonders Beschäftigte ohne viele private Kontakte im Home-Office zunehmend einsam und haben weniger Kontakt zu ihren Kollegen. Durch eine Korrelationsanalyse hat sich ebenso gezeigt, dass Beschäftigte im Home-Office ein geringeres Zugehörigkeitsgefühl zum Arbeitgeber und dem Rest des Teams entwickeln und durch ihre Abwesenheit seltener an Entscheidungen beteiligt werden. Umgekehrt formuliert ist also die erfolgreiche Vermeidung von sozialer Isolation ein zentraler, kritischer Erfolgsfaktor für das ideale Arbeiten im Home-Office. Es ist demnach dringend zu empfehlen entgegenwirkende Maßnahmen zu ergreifen, um Beschäftigte im Home-Office konsequenter miteinzubeziehen und soziale Berührungspunkte zu schaffen.

Ergänzend ist anzumerken, dass ideales Arbeiten im Home-Office nicht unmittelbar in jeder Hinsicht erreichbar ist und dies auch nicht sein muss. Die Einhaltung der grundlegenden Anforderungen, besondere Rücksicht auf die kritischen Erfolgsfaktoren sowie ein stetiger Verbesserungsprozess des eigenen Home-Office-Konzeptes sind das Ziel.

## Fazit

Für die Beschäftigten bestehen die wichtigsten durch das Home-Office entstehenden Effekte überwiegend in der zeitlichen Flexibilität, generellen Zeitersparnis und der damit einhergehenden besseren Vereinbarkeit von Familie und Beruf. Damit erfüllt das Home-Office mit den positiven Effekten auf die Beschäftigten viele Aspekte des New-Work-Konzeptes, bei welchem vor allem Werte wie die Freiheit, Zufriedenheit und Selbsterfüllung der Beschäftigten im Vordergrund stehen sollen. Auch Arbeitgeber können in vielen Aspekten von der Arbeit im Home-Office, wie in Kapitel 2.3 dargestellt, profitieren. Neben kostensparenden Effekten wie der Platzersparnis durch weniger benötigte Büroflächen, gibt es viele Effekte, die das zukünftige Bestehen des Arbeitgebers verbessern. So können Arbeitgeber mit dem Einsatz von Home-Office einen Digitalisierungsschub erleben und zeitgleich ihre Attraktivität als Arbeitgeber für potenzielle Beschäftigte steigern. Auch die Produktivität der Beschäftigten steigt im Home-Office an. Allerdings besteht an die Arbeitgeber ebenfalls eine große Anzahl an Anforderungen, um ideale Arbeit im Home-Office gewährleisten zu können. Die eigene Studie hat aufgezeigt, dass neben grundlegenden Anforderungen an die Arbeitsplatzausstattung, die Digitalisierung der Arbeitsprozesse und eine gute Organisation häufig individuelle Anforderungen in Hinblick auf die unterschiedlichen Lebenssituationen der Beschäftigten bestehen. Von den aufgeführten Anforderungen sind besonders die in Abschnitt 4.3 beschriebenen kritischen Erfolgsfaktoren zu berücksichtigen, da diese einen weitreichenden Einfluss auf das Gelingen eines Home-Office Konzeptes haben. Auch auf weitere Gruppen der Gesellschaft hat die Nutzung des Home-Office verschiedene Effekte. Dabei kann jedoch nicht abschließend festgelegt werden, ob diese überwiegend positiv oder negativ sind. Neben den in Kapitel 2.3 vorgestellten positiven Effekten wie einer geringeren Umweltbelastung oder einer alternativen Nutzung von Büroflächen, bewirkt das Home-Office bei bestimmten Branchen durchaus wirtschaftliche Rückgänge.

## Footnotes

1 Im vorliegenden Beitrag wird bevorzugt gendergerechte Sprache verwendet. Einzelne Begriffe können jedoch aus Gründen der besseren Lesbarkeit davon abweichen. Solche sind als genderneutral zu betrachten.
